# Supplementary material for: The structure of a 15-stranded actin-like filament from Clostridium botulinum
Source: Nat Commun. 2019 Jun 28;10:2856. doi: 10.1038/s41467-019-10779-9 (PMC6599009; doi:10.1038/s41467-019-10779-9)
Supplement: Supplementary file 1 — Supplementary Information [file 41467_2019_10779_MOESM1_ESM.pdf]

Supplementary Information

The structure of a 15-stranded actin-like filament from *Clostridium botulinum*

F. Koh et al.

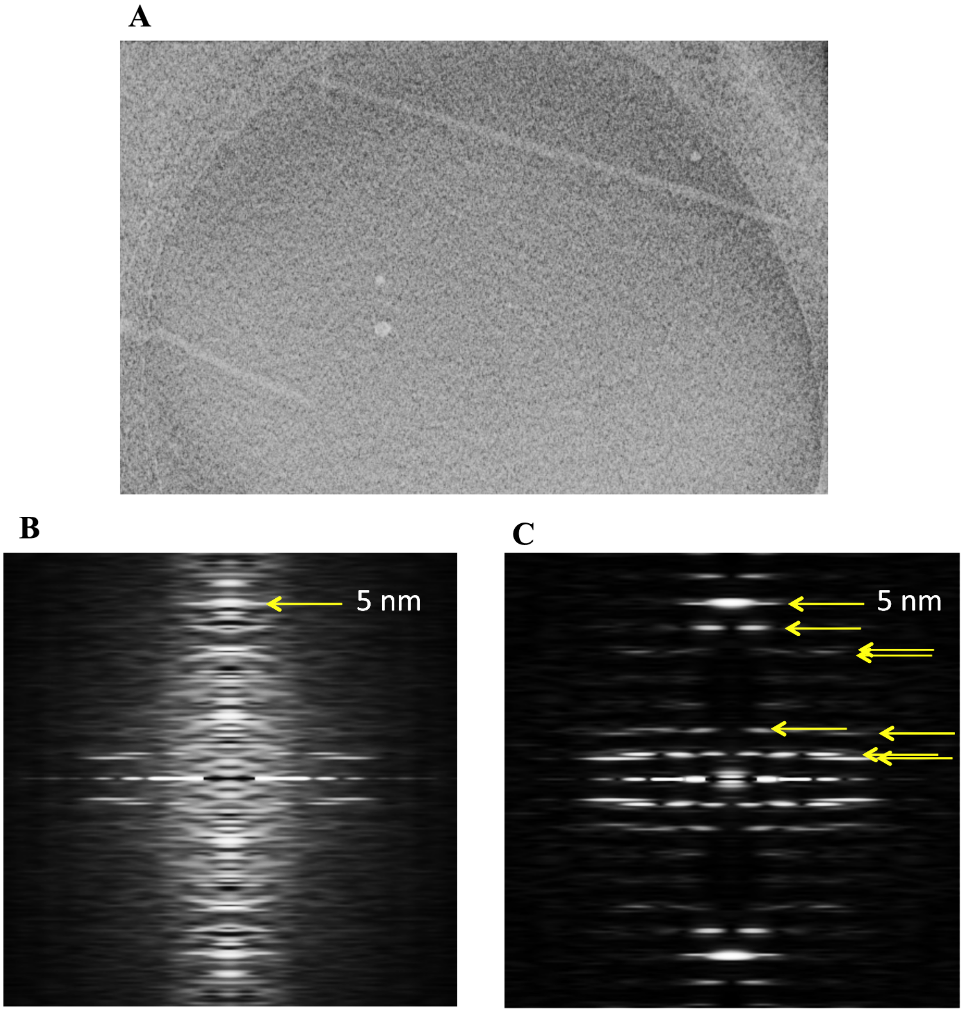

Supplementary Figure 1. pCBH ParM filament symmetry determined by Cryo-electron tomography. **a**, Cryo-electron tomogram of pCBH ParM filaments. **b**, A diffraction pattern from one tomogram. **c**, A diffraction pattern of a helically averaged tomogram (axial rise = 5.2 nm, helical twist -50.1 degrees). The yellow arrows indicate the similar layer lines as in Figure 2b.

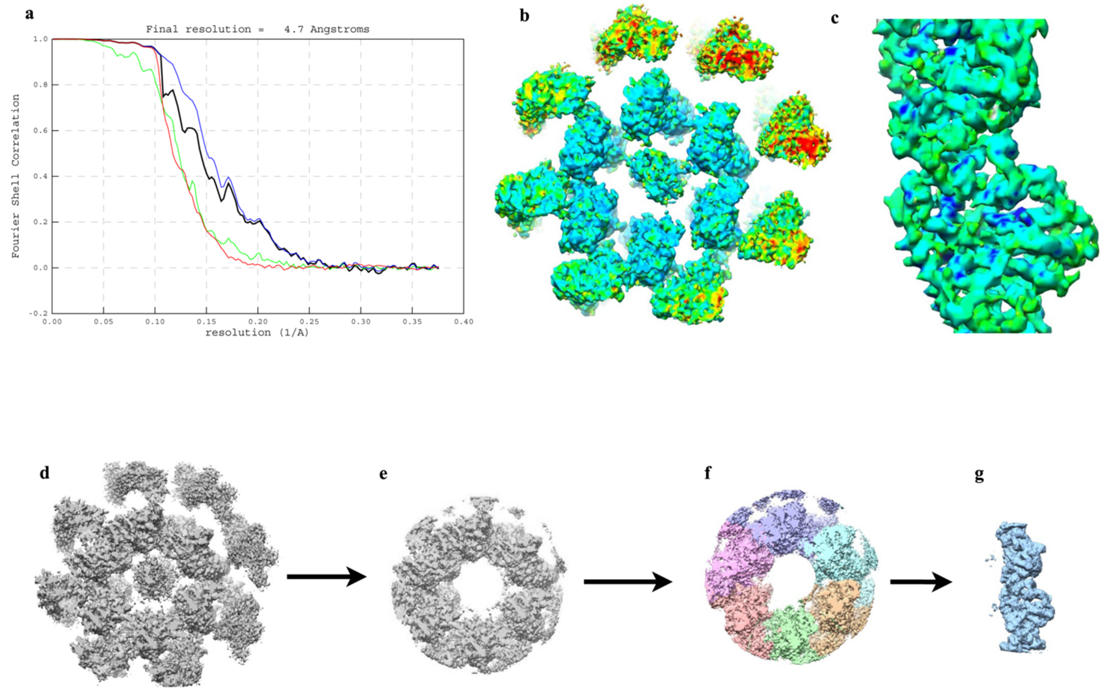

Supplementary Figure 2. Resolution and averaging of the pCBH ParM filament cryoEM data. **a**, Fourier shell correlations (FSCs) for evaluating the resolution calculated by RELION[14,15]. FSCs between two 3D structures from each half of the dataset with or without masking are presented in green and blue, respectively. The red curve was calculated after the phase was randomized beyond 8.6 Å to evaluate artefacts from overfitting[43]. The black curve is the corrected FSC after accounting for the artefacts from overfitting. Based on the golden standard criteria, the resolution is 4.7 Å with a threshold of 0.143. **b**,**c** Local resolution evaluated by ResMap[34]. **b**, Cross-section, Blue, cyan, green, yellow, orange and red indicate 3.6, 4.9, 6.2 7.4, 8.7 and 10 Å resolution, respectively. **c**, Averaged intermediate layer strand. Blue, cyan, green, and yellow indicate 3.0, 3.8, 4.6 and 5.4 Å resolution, respectively. (**d**-**g**): Real space averaging of the six strands in the intermediate layer. **d**, The whole map. **e**, The extracted intermediate layer. **f**, Segmented strands. **g**, Averaged strand.

a

```
                                                                                       A1                                                                  A2
AJD29063.1     1  MNKYTIAIDLGYGQIKGINQDNKRVIFPSIISSGKDRSLDTFFNSIDNIVDNIHVKILDE
Mutant         1  MNKYTIAIDLGYGQIKGINQDNKRVIFPSIISSGKDRSLDTDFNSDDNIVDNIHVKILDE

AJD29063.1    61  YFNEKEYFVGELAKRQPSNSSFINRDNKINSEENKVLLATALGLLIPNDLPNDTKIHIVT
Mutant        61  YFNEKEYFVGELAKRQPSNSSFINRDNKINSEENKVLLATALGLLIPNDLPNDTKIHIVT

AJD29063.1   121  GLPLEHFIKQKQALNDMLKDFEHTIKFVDHNFSRNIKFEESNITLFPQGAGAIFSKINND
Mutant       121  GLPLEHFIKQKQALNDMLKDFEHTIKFVDHNFSRNIKFEESNITLFPQGAGAIFSKINND

AJD29063.1   181  ISSLLIKETFIGLIDVGFKTTDIVVFRINKDKEPVFEQEMSATLDGLGMINIYNTMDKAF
Mutant       181  ISSLLIKETFIGLIDVGFKTTDIVVFRINKDKEPVFEQEMSATLDGLGMINIYNTMDKAF

AJD29063.1   241  TDNSRDGSKLNTEQLMLLCEEGKIFFKGDYIDLKKDLIKARKTLSTNIINKADGLWGSRK
Mutant       241  TDNSRDGSKLNTEQLMLLCEEGKIFFKGDYIDLKKDLIKARKTLSTNIINKADGLWGDDK

AJD29063.1   301  NSFNSIMIAGGGGKVLYNHLKLIEPNMCQLIDNPEFANAIGYLEFGKQFK
Mutant       301  NSFNSIMIAGGGGKVLYNHLKLIEPNMCQLIDNPEFANAIGYLEFGKQFK
```

b

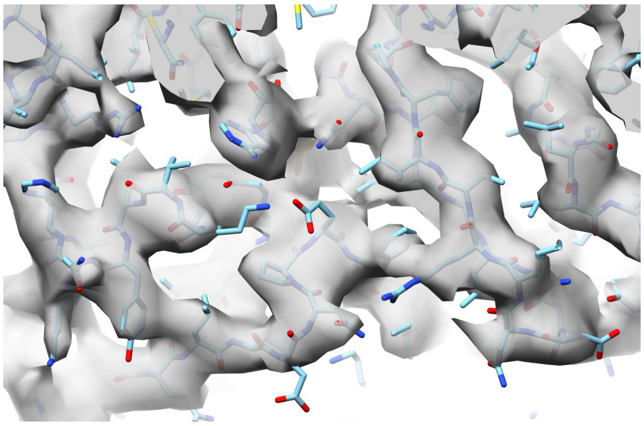

c

CATATCAAATAACCTTATAACTAGCTAATATTTAATTAAAAACTATATATATTAGCTAG
TTATTTATATTCTGATTTGTATACATTTAATAAAATACATGTAAAGCTAGCCATATAAG
TATATACAGTACATTATGTATACAACATATGTGTACATATTATATTTTTCATATTTTTAA
TTAATCATTACATATGGTTCTAAGTTGTATACAATGAATGTAATACATATCAGTCTAG
CTATATTAATAACTTTTTACAAAATTGTATACAGTATATGTATACATATCTCTATGAAA
TAGTATTTCCTAATAAAAATGTATACGAATCTTTTAAAAAGATATCATTATTTTTCTTT
TGCATACACAAATTGTATTACTAACTAATGTATATATACTAATAATATTTACTACATTG
TATACAGTATATGTATACATATTGAAAAATGTACACCTACATTCTGGAAGAAGTTCTG
AAA

Supplementary Figure 3. Sequences of pCBH ParM and pCBH *parC*. a, The pCBH ParM sequence is aligned with the mutant sequence used for crystallography. Marks above the sequences highlight important residues: Ball-and-socket residues (cyan and pink stars; Fig. 7a); Long chain residues (yellow and gray diamonds, Fig. 7a); Magnesium ion binding residues (green crosses, Supplementary Figure 4); and acidic loops (red bars, Fig. 3b and Supplementary Figure 4). **b**, Example of the cryoEM density with the model fitted with sidechains. **c**, The pCBH *parC* sequence used in Fig. 1d.

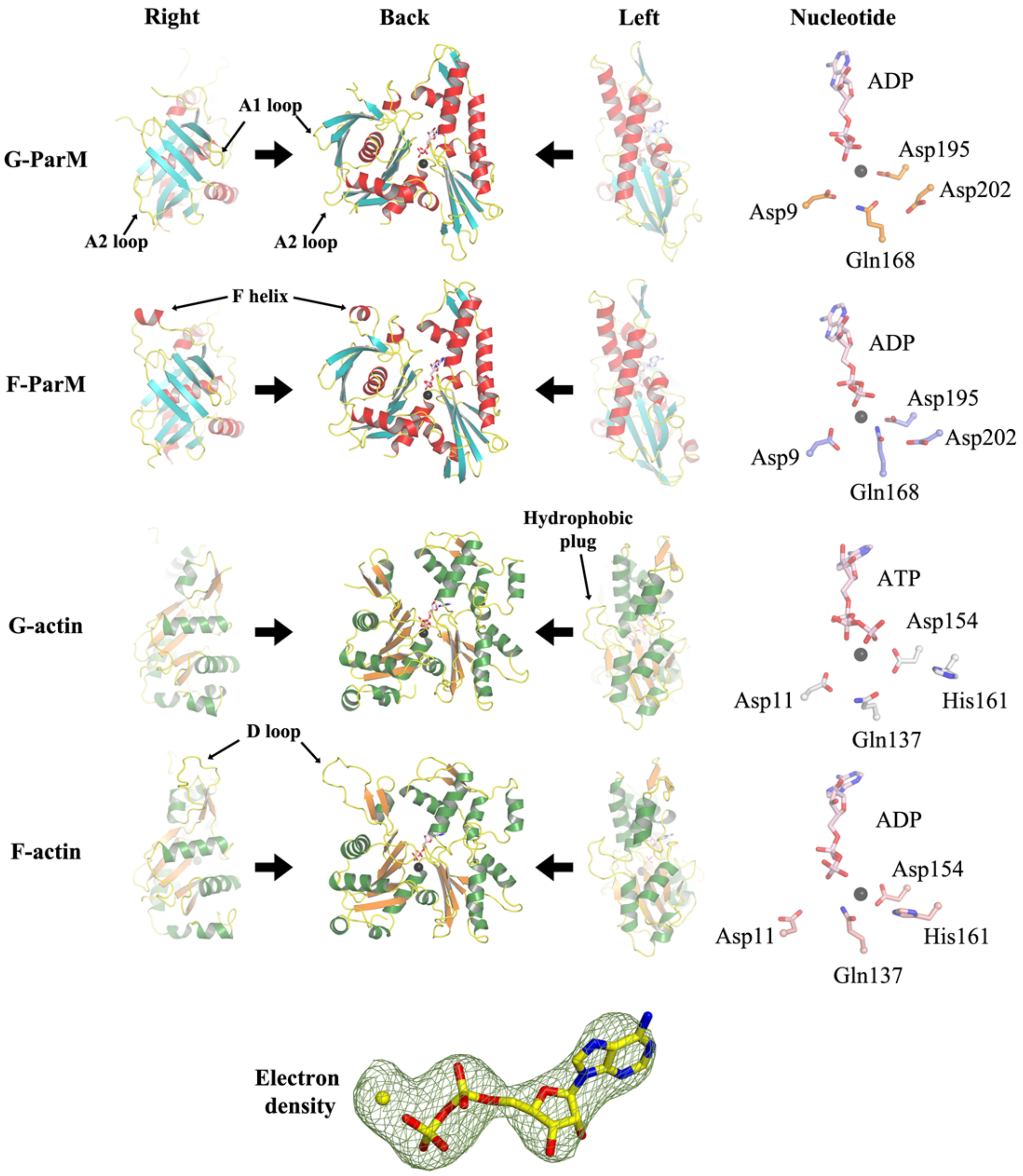

Supplementary Figure 4. The pCBH ParM protomer. Different views of G-ParM, F-ParM, G-actin and F-actin, relative to Fig. 3b-e, and the configuration of their nucleotide and cation binding sites. In G-actin, the interactions of Asp11, Asp154 and Gln137 with the cation is mediated through water molecules (not shown). The resolutions of the G-ParM, F-ParM and F-Actin structures are not sufficient to place the corresponding water molecules. The average composite OMIT map around the pCBH ParM bound ADP is shown contoured at 1σ.

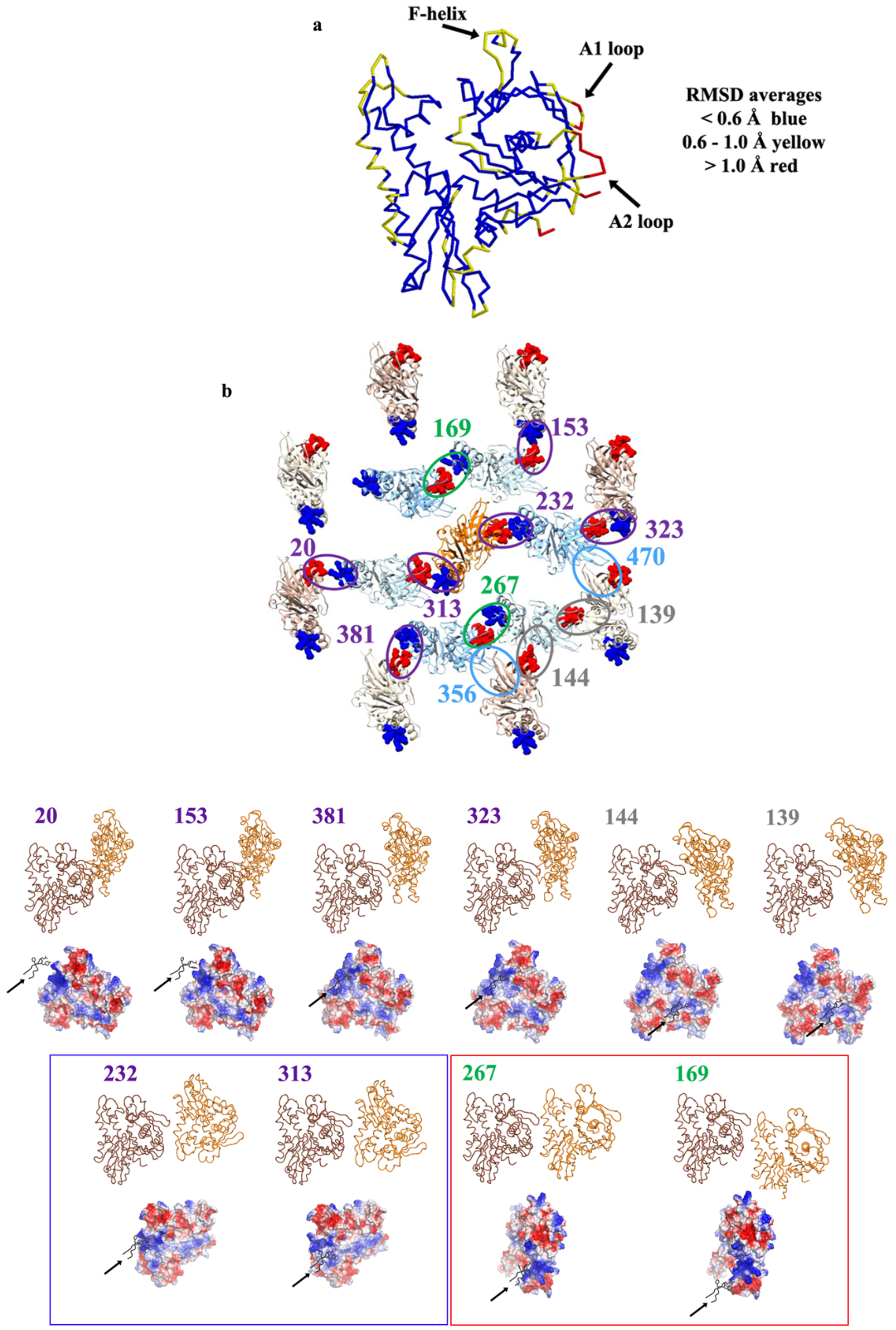

Supplementary Figure 5. Interactions within a pCBH ParM cross-section. **a**, Comparison of RMSDs of different regions of the pCBH ParM protomer conformations from the filament. **b**, Interactions within a pCBH ParM cross-section. Top panel indicates the inter-strand contact areas between protomers (Å^2) in a single cross-section. Lower panels show inter-strand contacts that involve the first acidic loop (A1 loop), indicated by the arrows. The interactions are labeled by their contact areas. Blue box, antiparallel central-intermediate layer interactions. Red box, parallel intermediate-intermediate layer interactions. The remainder are antiparallel outer-intermediate layer interactions.

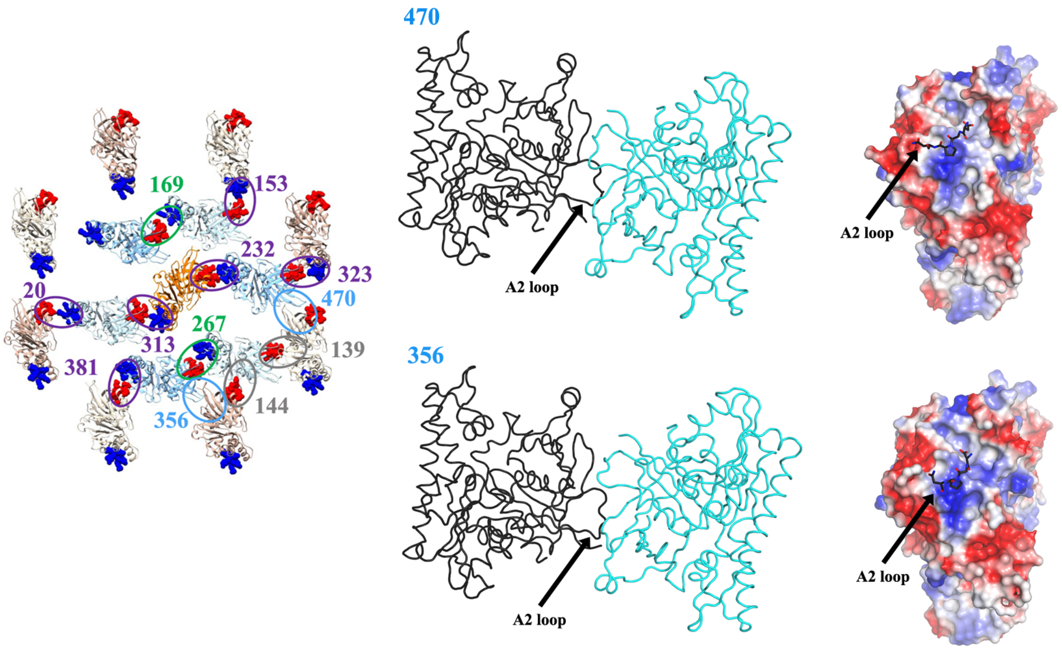

Supplementary Figure 6. Interactions within a pCBH ParM cross-section involving A2 loops. Left panel indicate the inter-strand contact areas between protomers (Å^2) in a single cross-section. Central panel shows the two contacts that involve the second acidic loop (A2 loop). The interactions are labeled by their contact areas. Left panel show the contact of the A2 loops with the interacting protomer (shown as charge surfaces).

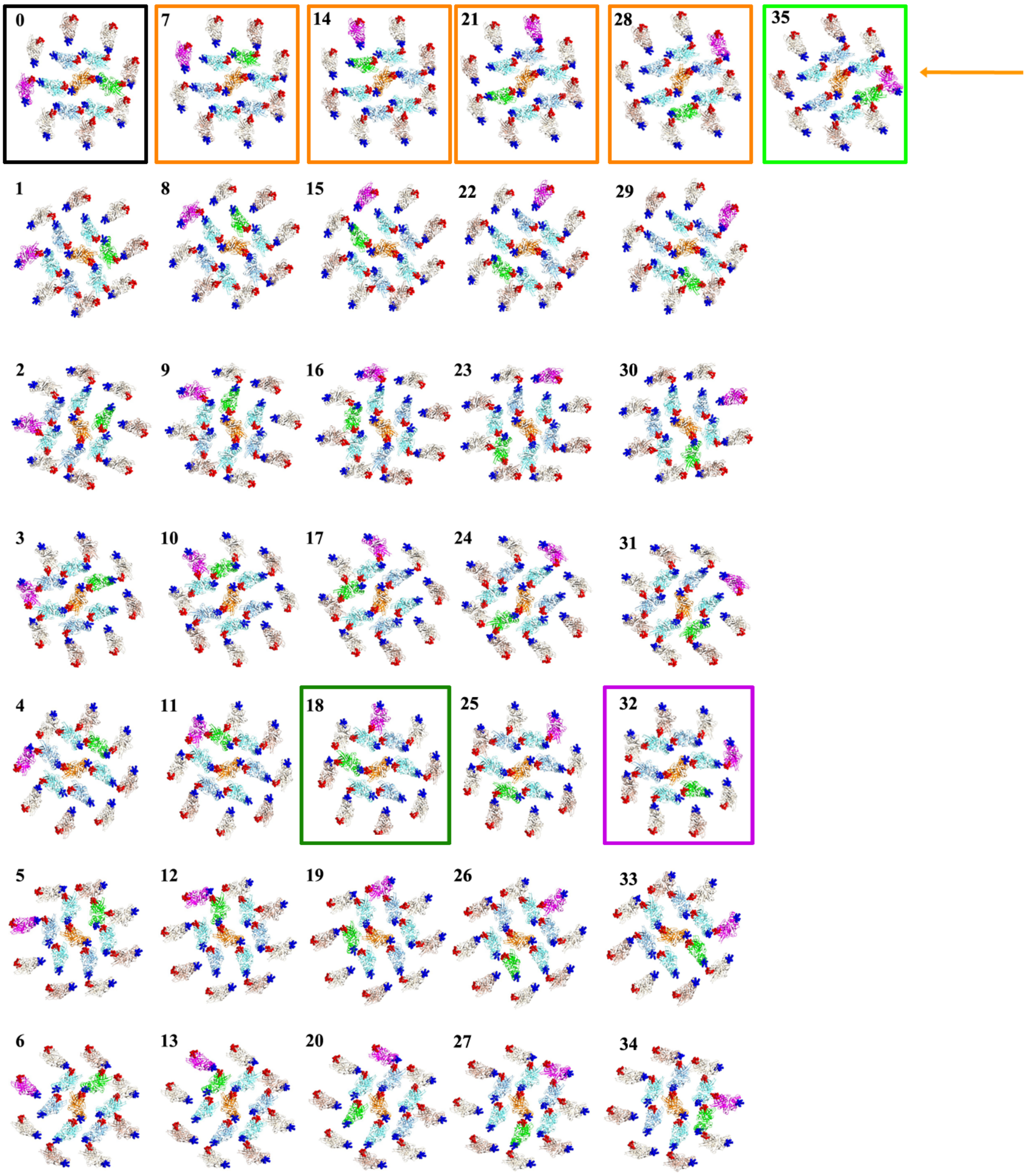

Supplementary Figure 7. The repeating unit of the pCBH ParM filament. Numbers indicate subsequent cross-sections in moving towards the barbed end of the central filament. The central filament repeat is ~7 protomers. The orange arrow indicates the cross-sections where the central protomer is in similar orientations. A path of a strand in the intermediate layer rotates by 360 degrees around the center axis in the span of 37 protomers. Cross-sections 0 and 18 have the green protomer on opposite sides of the filament (half a repeat, dark green box). Cross-sections 0 and 35 have the green protomer in a similar position (light green box). The outer layer rotates by 360 degrees in the span of 67 protomers. Cross-sections 0 and 32 have the pink protomer on opposite sides of the filament (half a repeat, pink box). The cross-sections are shown as a time lapse movie (Supplementary Video 2).

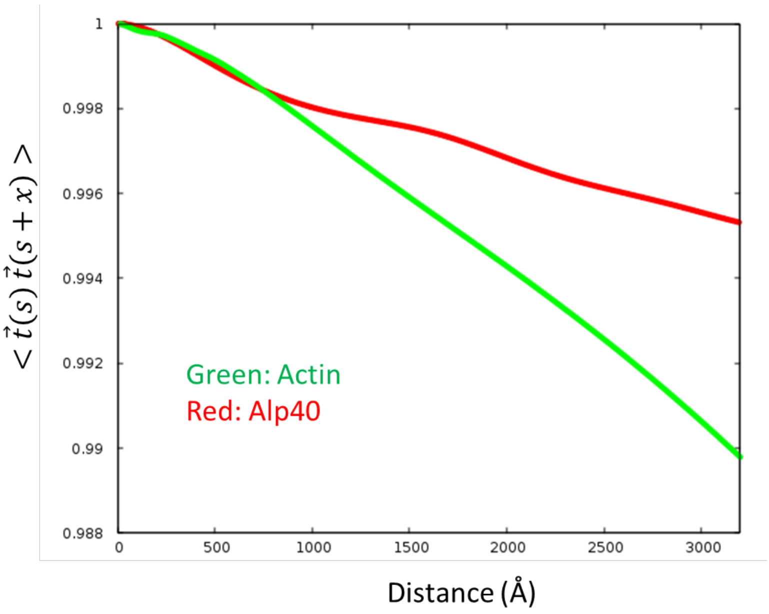

Supplementary Figure 8. Persistence lengths of the pCBH ParM filament and actin filament. The average of inner product, $\vec{t}(s) \cdot \vec{t}(s+x)$ is plotted against distance x. The persistent length (Lp) was calculated by fitting $< \vec{t}(s) \cdot \vec{t}(s+x) >= \exp\left(-\frac{x}{2L_P}\right)$. The persistent length was 35 µm for the pCBH ParM filament and 11 µm for the actin filament.

Supplementary Table 1. X-ray data collection and refinement statistics.

	pCBH ParM (PDB 3IXW)
**Data collection**	
Crystal	C2
a, *b*, *c* (Å)	164.2, 93.90, 114.7
α, β, γ (°)	90.0, 131.6, 90.0
Wavelength (Å)	1.0
Resolution (Å)	20.0-3.25 (3.31-3.25)
R_{merge}	14.3 (57.4)
R_{meas}	17.0 (69.6)
R_{pim}	9.1 (38.8)
$I/\sigma(I)$	6.8 (1.9)
$CC_{1/2}$	0.965 (0.562)
Completeness (%)	99.9 (99.1)
Redundancy	3.5 (3.2)
**Refinement**	
Resolution (Å)	20.0-3.25 (3.42-3.25)
No. reflections	19789 (2269)
R_{work} / R_{free}	15.8/21.4 (20.6/27.0)
No. atoms	
Protein	8111
Ligand/ion	126 (ADP)/5 (Mg^{2+})
B factors ($Å^2$)	
Protein	60.3
Ligands	61.5
r.m.s deviations	
Bond lengths (Å)	0.010
Bond angles (°)	1.26
Ramachandran Plot	
Favoured (%)	94.1
Outliers (%)	0.3

Values in parenthesis refer to the parameters for the highest resolution shell.

Supplementary Table 2. CryoEM data collection, refinement and validation statistics

	All strands (EMDB-9757) (PDB 6IZR)	Intermediate strand (EMDB-9758) (PDB 6IZV)
**Data collection and processing**		
Magnification	91 k	91 k
Voltage (kV)	300 kV	300 kV
Electron exposure (e–/Å^2)	30	30
Defocus range (μm)	1.5~3.5	1.5~3.5
Pixel size (Å)	1.331	1.331
Symmetry imposed	Helical	Helical
Initial particle images (no.)	36293	36293
Final particle images (no.)	33356	33356
Map resolution (Å)	4.7	4.2
FSC threshold	0.143	Mean value of local resolution
Map resolution range (Å)	∞ ~ 4.7	∞ ~ 4.2
**Refinement**		
Initial model used (PDB code)	6IZV	6IXW
Model resolution (Å)	4.7	4.2
FSC threshold	0.143	Mean value of local resolution
Model resolution range (Å)	∞ ~ 4.7	∞ ~ 4.2
Map sharpening *B* factor (Å^2)	-175	-175
Model composition		
Non-hydrogen atoms	83500	5568
Protein residues	10470	698
Ligands	60	4
R.m.s. deviations		
Bond lengths (Å)	0.005	0.005
Bond angles (°)	1.123	1.109
Validation		
MolProbity score	1.75	1.66
Clashscore	5.60	5.77
Poor rotamers (%)	0.00	0.00
Ramachandran plot		
Favored (%)	93.1	95.1
Allowed (%)	6.9	4.9
Disallowed (%)	0	0

Supplementary Table 3. Primer sequences

**PRIMERS FOR PRODUCING LINEAR PARC FROM THE PUC57 PLASMID**	
5' 6-FAM-M13-FW	5' 6-FAM-CCCAGTCACGACGTTGTAAAACG
M13-RV	AGCGGATAACAATTTCACACAGG
**PRIMERS FOR MUTATING PARM FOR CRYSTALLIZATION**	
CBM40_F42D_147D_F	CAAAGATCGTTCACTGGACACCGATTTCAACTCGGACGATAACATCGTGGATAACATC
CBM40_F42D_147D_R	GATGTTATCCACGATGTTATCGTCCGAGTTGAAATCGGTGTCCAGTGAACGATCTTTG
CBM40_S298D_R300D_F	CAAAGCCGACGGCCTGTGGGGTGACGACAAAAATAGCTTTAACTCTATTA
CBM40_S298D_R300D_R	TAATAGAGTTAAAGCTATTTTTGTCGTCACCCCACAGGCCGTCGGCTTTG
CBM40_F42D_147D_F	CAAAGATCGTTCACTGGACACCGATTTCAACTCGGACGATAACATCGTGGATAACATC
