# Supplementary material for: The structure of a 15-stranded actin-like filament from Clostridium botulinum
Source: Nat Commun. 2019 Jun 28;10:2856. doi: 10.1038/s41467-019-10779-9 (PMC6599009; doi:10.1038/s41467-019-10779-9)
Supplement: Supplementary file 3 — Description of Additional Supplementary Files [file 41467_2019_10779_MOESM3_ESM.pdf]

Description of Additional Supplementary Files

File name: Supplementary Movie 1

Description: The architecture of the pCBH ParM filament

File name: Supplementary Movie 2

Description: The cross-section layers of the pCBH ParM filament
